# Oil and renewable energy returns during pandemic

**DOI:** 10.1007/s11356-022-23903-y

**Published:** 2022-11-08

**Authors:** Florian Horky, Mihai Mutascu, Jarko Fidrmuc

**Affiliations:** 1 Zeppelin University, Friedrichshafen, Germany; 2 West University of Timisoara, Timișoara, Romania; 3 Laboratoire d’Economie d’Orleans (LEO), University of Orleans, Orleans, France; 4 Faculty of Business and Economics, Mendel University in Brno, Brno, Czech Republic; 5 East-European Center for Research in Economics and Business (ECREB), West University of Timisoara, Timișoara, Romania; 6 Institute of Economic Research, Slovak Academy of Sciences, Bratislava, Slovakia

**Keywords:** Oil energy, Renewable energy, Pandemic crisis, Wavelet analysis, C14, E24, O40

## Abstract

We explore the global interactions between oil and renewable energy returns during the Covid-19 pandemic between July 2019 and June 2020. Moreover, we reflect on market stress and global economic activity. In order to deal with challenges generated by exogenous shocks coming from financial, economic or pandemic areas, a battery of advanced time–frequency domain methods is applied, ranging from wavelet transformation and wavelet coherency to wavelet cohesion. The main finding shows that pandemic disease is veritable glue for the oil energy–renewable energy nexus, validating their coupling effect. Additionally, the emerging connection between renewable and financial developments is evidenced during the pandemic crisis, although the connection between oil and financial developments is still stronger. Finally, both renewable energy and oil markets have comparably strong relationships with the general global economic activity. The policy implications should follow direct adjustments in the renewable energy area, and subsidiary to cover the behaviour of agents on the capital markets.

## Introduction

The Covid-19 pandemic has had a massive impact on the entire economy, the financial markets and thus also the energy markets. Since the latter take on a special role in overcoming social and environmental challenges, they are of particular interest. Furthermore, there is an ongoing shift away from fossil fuels towards renewable energies in progress, which is further strengthened by the war in Ukraine, which started in February 2022. Thus, the price of oil has come under pressure during the pandemic, especially in the course of the crisis due to lower consumption, for example as a result of the massive slump in international travel (Sharif et al. 2020). However, the renewable energy sector has also suffered massively from the supply chain constraints of the Covid-19 pandemic and initially unclear policy responses (Eroğlu 2020). Furthermore, the sanctions and supply barriers due to Ukraine, despite soaring energy prices, seem to be supporting a move away from traditional energy resources towards renewable sources. In this context, the paper analyses an actual and important topic as the Covid-19 pandemic shock unveils new complex challenges regarding the transition from conventional to renewable energy.

The oil market has been an important traditional indicator of overall economic development. Oil price shocks, e.g. due to a changed pricing policy of oil-producing countries, have had a massive impact on the stock markets. There are still large spillover effects between the financial and energy markets in terms of volatility and shocks in one of the markets (Nazlioglu et al. 2015). However, this interrelation changed over time, so the contribution of supply shocks in the oil market to real economic activity has decreased significantly since the 1970s (Kang et al. 2015). The declining importance of the oil price for the overall economy is also connected with the increasing relevance of alternative and renewable energy sources in recent times. Nevertheless, the oil price is still the key commodity for the energy markets. Although renewable energy stock returns are highly sensitive to volatility shocks in the oil market (Dutta 2017), the link between crude oil markets and green energy stocks seems to be gradually diminishing, which would indicate a paradigm shift in the global energy markets (Ferrer et al. 2018).

The economic slump during the pandemic is likely to represent the major shock in recent history, overshadowing even the financial crisis of 2007–2008. Considering that renewable energies have only been on the rise in the past decade, both socially and politically, one can speak of the first big shock that hits the decoupled energy markets. This development raises special interest in light of the latest events around the climate debate regarding the Paris climate agreement and the Fridays for Future movement. Especially the latter, as a youth movement with several million participants worldwide, is driving the public debate.

Due to this background, it seems particularly interesting to examine these two poles – the oil market and the market for renewable energies – of the global energy market and their connection to financial stress levels in the wake of the Covid-19 pandemic. Not at least, a correct understanding of the interaction between the stock prices of traditional energy companies and the stock prices of renewable energy companies reduces the information asymmetries for all market participants but also offers useful information helping the policymakers in their decision.

In this light, the main aim of the paper is to investigate the nexus between the crude oil market and the renewable energy market from a stock market perspective before, during and shortly after the peak phase of the Covid-19 pandemic on the stock markets. The pandemic crisis offers an excellent research environment, as it is possible for the first time to simultaneously examine the effects of an exogenous shock affecting the entire economy on renewable energy markets as well as the oil markets. The novelty of the approach also comes from the accent put on the decoupling effect between oil energy and renewable energy markets under this pandemic episode.

In order to conduct our analysis, we use a spectrum of modern wavelet methodologies covering the period from July 2019 to June 2020. The time period is selected in order to ensure the all-inclusive approach while maintaining an optimal quality of wavelet plot details (i.e. avoiding loss of details as the number of observations increases). For this analysis, we use two stock market indices, covering stocks of oil-producing companies (XOI) and companies in the renewable energy sector (RENIXX). We complement this data with data on financial stress and Google Trends data considering the Covid-19 pandemic.

The theoretical ground is given by the contribution of Lee and Baek (2018), who link the oil energy market with the renewable energy market by indicating any extension of the oil sector being associated with an increase in share prices of renewable energy firms as a result of competitive advantage. Additionally, such a connection is strongly impacted by volatility spillover effects between the oil market and renewable energy stocks under various shocks (i.e. pandemic disease), showing that they grow or crash together (Song et al. 2019; Reboredo 2015).

One important difficulty in the environmental field investigations is to accurately filter the exogenous shocks’ various origins, such as those coming from financial, economic or pandemic areas. Fortunately, this challenge can be successfully overcome by following a time–frequency empirical approach by wavelet type.

The wavelet modelling offers excellent results as our targeted topic supposes data with high volatility and different shocks, such as those registered in the environmental and energy areas. By locating the data both in time and frequency, the developed model has the capacity to directly capture those breaking points, with high accuracy, over different sub-periods of time, without any additional adjustments (i.e. threshold effects, dummy variables, span splitting).

The contribution of this paper is threefold, covering the idea of a decoupling effect between oil and renewable energy markets across different sub-periods of times and frequencies under specific pandemic, financial and economic contexts.

Therefore, the paper is one of the first analyses focusing on the decoupling effect between oil and renewable energy resources before and during pandemic crisis. In other words, with this timeframe, we cover a period which allows us to compare the energy markets’ behaviour before, during and shortly after the crisis. Comparing Magazzino et al. (2021), Mutascu et al. (2022), Mutascu and Sokic (2020) and Mutascu (2018) which treat the issue of gas emissions, oil prices co-movements or energy consumption related to trade openness or economic growth by using similar wavelet tools, this paper adds as a novelty the particular episode of Covid-19 pandemic disease in the environmental field. Unlike existing papers, the study connects oil energy with renewable energy by considering the pandemic shock under financial stress and global economic worldwide context.

Second, both financial and economic determinants are considered to control the oil–renewable energy interactions. Such factors are crucial for the oil–renewable energy co-movement, being very sensitive across different bands of frequency.

Third, unlike the major part of studies in the field which use classical econometric time-domain methods, our paper follows the wavelet approach. This tool is superior to the classical ones, offering information not only about sign, intensity and direction of co-movement between variables but also about their developing evolvement at different frequencies over time (Pomenkova et al. 2019). It is noteworthy that the wavelet has the propensity to expand those insights in the short, medium and long runs, perfectly fitting the sensitivity across various frequencies of oil–renewable energy nexus.

The institutional promotion of renewable energy, investors’ diversification of portfolio risk, reduction of negative publicity by following renewable energy and improvement of basic liquidity of renewable energy firms underline the relevance of policy implications. The main research limit of the study is given by the fact that no extended pallet of determinants is considered due to a lack of official data availability, especially regarding pure economic indicators.

The remainder of this paper is organised as follows: The “Literature” section presents the literature review; the “Data and methodology” section describes the methodology and data, while the “Findings” section shows the empirical findings. Furthermore, the “Robustness check” section reveals the robustness checks, and the “Conclusions” section concludes.

## Literature

The interlinked global energy markets are in a constant process of change. Technological progress, geopolitical conflicts and political endeavours have shaped and continue to shape the development of the energy sector (Huppmann and Egging 2014). Thereby, energy consumption is a determining factor for economic growth and prosperity. For example, Magazzino et al. (2021) explore the interaction between energy consumption and growth in Italy by using the wavelet methodology. They find mixed results, with the influence of energy consumption on economic growth being observed only at low scales (i.e. long run). At the same time, oil price plays an important role in conventional energy production as well as the interaction between prices of different types of derived products. In this vein, Mutascu et al. (2022) investigate Germany, France and Italy from January 2005 to June 2021 by following a time–frequency approach. They evidence a significant link between gasoline and diesel prices at all frequencies, becoming stronger in the long run. Moreover, despite different applied fuel tax systems, no influence on the co-movements of fuel prices is observed between gasoline and diesel.

However, society has reached a point where the energy sources used must be balanced in terms of not only their economy but also their ecological impact (Sadorsky, 2009). Under the influence of the Paris Climate Agreement, the Fridays for Future movement and geopolitical uncertainties, a transition process towards renewable energies can be observed. This effect also manifests itself in different uses of various energy sources. Oil as an energy carrier is mainly used for transport services, while renewable energies are used for electricity production.

This has been accompanied by a decoupling of the markets for renewable energies and oil (Ferrer et al. 2018) at least in the middle of 2010s. When investigating the oil price plunge in 2014, Khan et al. (2017) find no significant impact on the renewable energy sector, which can be easily explained by the above-mentioned mechanisms. From a historical perspective, the most relevant drivers for this transition process are both the technological production possibilities and the associated prices, combined with a corresponding political-systemic environment. In this vein, many researchers intensified their work to design optimal control strategies for a standalone hybrid power system (HPS). For example, Tahiri et al. (2021) propose a complex tool ranging from photovoltaic system (PVS) and wind energy conversion system (WECS) to a pulse width modulation (PWM) voltage source inverter (VSI). Unlike them, Qerimi et al. (2021) develop a system able to replace conventional water heaters with domestic solar water heaters (DSWH).

However, crises and energy price shocks can act as accelerators in this process (Fouquet 2016). In any case, it is to be expected that the diffusion of renewable energies into the world energy mix will increase the complexity of the geopolitical energy markets (Hache, 2018). This is accompanied by the urgent need to uncover interrelations between the individual energy markets, for example in crisis behaviour, in order to be able to act politically accordingly.

At the same time, energy, or its carriers, has always been an important commodity on the international stock exchanges and is therefore subject to speculation. In this light, it seems obvious that a strong link between the energy markets and financial markets has been established over time (Nazlioglu et al. 2015; Elsayed et al. 2020; Demirer et al. 2020). The most important medium of energy, traded on the international markets in recent decades, is crude oil, with the connection of its price to financial stress being subject to extensive research. Here, we want to present this first strand of literature with its most important findings on the basis of selected studies. Cunado and de Gracia (2014) investigate the connection between oil prices and stock market returns in 12 European countries, identifying a significant negative reaction of stock returns to oil price shocks. Baumeister and Kilian (2016) see the financial crisis of 2007–2008 as a powerful demonstration of the effects of a sharp, financially induced drop in demand for fossil energy carriers on the price of these. In this way, they propose a transmission of financial stress towards the oil price due to a demand channel which is in line with Chen et al. (2014), where they find a significant transmission of financial shocks, leading to declining oil prices. They also note the increasing financialization of the commodity markets.

However, the exact form of the relationship between financial and oil markets is the subject of investigation and debate. Some studies suggest a significant but nonlinear relationship between stock market returns and oil price, or in some cases, oil price futures (Ciner 2001; Zhang 2017; Yao and Kuang 2019). Ciner (2001) additionally points out that this relationship changes over time, with a peak in connectedness in the 1990s. Nevertheless, there is widespread agreement that the underlying cause of a shock change in oil prices, i.e. supply-induced or demand-induced, is significantly important, considering the consequences (Cunado and de Gracia 2014; Kilian and Park 2009; Demirer et al. 2020). The transmission effects between energy markets and financial markets can therefore be assumed not to be limited to equities but actually affect the entire financial market. Nevertheless, there are also critical contributions regarding the importance of crude oil prices and fluctuations in the stock market returns. Elsayed et al. (2020) support the idea of a strong connectedness between energy markets and stock markets. However, they do not find evidence for the oil price having a key impact on stock market fluctuations in the period from 2000 to 2018. Reboredo and Uddin (2016) only find weak evidence for Granger causality and co-movement between general stock market uncertainty and commodity futures pricing. The results of Yao and Kuang (2019) provide a good summary of the literature before the pandemic. They find a relationship between stock markets and oil markets, but this relationship changes over time. Furthermore, their results show that spillover effects between crude oil prices and stock markets differ across developed and emerging markets.

Next to fossil fuels, renewable energies have experienced a strong upswing in recent years. Compared to fossil fuels, the connection between renewable energies and the financial markets is still relatively unexplored. This may also be due to the fact that renewable energies are much more difficult to grasp than fossil fuels, i.e. there are still hardly any tradable storage media for renewable energies. Wei et al. (2022) suggest that renewable energy markets are quite heterogenous and reflect extreme climate risks, forcing investors to diversify their respective portfolios. For this reason, renewable energies are proxied on the stock exchanges in the form of shares of green energy companies and not as classic commodities. These green energy stocks, therefore, entail characteristics of both the general stock market and traditional commodities (Shahzad et al. 2020). The available literature, however, allows the comparably strong conclusion that the development of renewable energies is strongly related to highly developed financial markets (Al Mamun et al. 2018; Kim and Park 2016). Particular emphasis is placed on access to capital, which is especially important for the respective complex technologies. Conversely, it is becoming increasingly important for investors in the financial markets to include social and ecological aspects in their investment decisions (Shahzad et al., 2020). This can be done via Green Bonds, whereby renewable energies also have an impact not only on the stock market but also on the entire financial market (Reboredo 2018). To summarise this first strand of literature, it can be concluded that energy markets, whether fossil fuels or renewables, have a strong link to the financial markets. However, the exact connections are the subject of continuous development with both economic and political roots. It seems as if the two markets not only influence each other but also react together to the underlying causes, such as political conflicts in the Middle East (Kilian and Park 2009) or during the Covid-19 pandemic.

Another strand of literature addresses the connection between the stocks of renewable energy firms and oil prices. Intuitively, rising oil prices are often associated with rising share prices of renewable energy firms, as the higher oil price creates a competitive advantage for renewable energies (Lee and Baek 2018). Conversely, a decline in oil prices would have a negative impact on the performance of renewable energy stocks, either directly or indirectly, due to an underlying decline in energy demand. The connection between the oil price and the returns of renewable energy stocks is the subject of numerous studies. Song et al. (2019) find significant volatility spillover effects between the oil market and renewable energy stocks and therefore propose a strong link between the two energy markets. Reboredo (2015) supports this connection by proposing that oil and renewable energy markets grow and crash together. In his study for the period from 2005 to 2013, he finds strong evidence for a significant contribution of oil prices to the systemic risk of renewable energy stocks. In a wavelet-based approach, Reboredo et al. (2017) re-examined this connection for the period from 2006 to 2015 and found a dynamic interaction, especially for low frequencies. Finally, Lee and Baek (2018) find asymmetric short-term effects of oil prices on the stock prices of renewable energy firms. However, there are also alternative voices, especially when considering the latest developments. Ferrer et al. (2018), for example, see increasing financialization of the commodity energy markets since the beginning of the 2000s, as well as a decoupling of the alternative energy market from the traditional energy markets. This leads to a fragmentation of the energy markets with individual dynamics. Zhang and Du (2017) find that the stocks of new energy companies correlate highly with high-tech company stocks and less with oil company stocks. In this sense, renewable energy stocks and fossil energy stocks are competing assets, whereby positive developments in renewable energy stocks positively affect the attractiveness of fossil fuel stocks (Wen et al. 2014).

During the Covid-19 pandemic, renewable energies, the oil price and the stock markets all suffered severe losses. The pandemic crucially affects the electricity and petroleum demand, whereby renewable energies, e.g. solar electricity, are less affected due to their decentralised characteristics (Norouzi et al. 2020).

Several literature gaps can be identified. The first gap is that no paper treats the coupling effect between oil and renewable energy during a pandemic disease by using a complex methodology such as the wavelet technique. The second gap shows that no authors used a mix of financial stress and global economic determinants as the main ingredient for the aforementioned nexus. Finally, no study investigates the situation before, during and shortly after the crisis’ first wave. All those gaps are addressed in the paper.

## Data and methodology

### Data

Two variables are used to investigate the co-movement between oil energy and renewable energy. The oil energy is captured through the XOI index, while the renewable energy is captured via the RENIXX index. Both indexes were retrieved from Thomson Reuters Eikon. The daily dataset covers the period July 01, 2019–June 26, 2020 (i.e. 217 observations). The period is focussing on the initial situation, in which both the first wave and the summer break are assessed in order to highlight the main ‘behavioural’ differences induced by those turbulences in the global energy market. Nevertheless, the period may be highly interesting also from the perspective of the Ukraine war because the developments of energy prices were nearly opposite. However, sanctions and supply constraints work in a similar direction to low energy prices during the pandemic. However, the data for Spring 2022 do not yet allow a deep analysis of the renewable and traditional energy nexus. Therefore, we focus on the pandemic episode here.

The oil energy is captured through the XOI index. It represents the NYSE Arca Oil Index as a price-weighted measure of the exploration, production and development of petroleum by the leading companies in the oil industry.

The proxy for renewable energy is the Renewable Energy Industrial Index (RENIXX). The index measures the renewable energy industry worldwide, taking into account the market capitalization for the 30 largest companies in the renewable industry (i.e. wind energy, solar energy industry, hydropower, geothermal energy, bioenergy or fuel cell technology).

A set of several variables is also considered to analyse the effects of pandemic signals, market stress and global economic activity on the oil and renewable energy markets.

The pandemic signal is obtained as a trend dimension generated by the Google Trends Index of coronavirus topic searching. The scale ranges between 0 and 100; the pandemic signal being stronger as the index value tends to be close to 100.

The Financial Stress Index of the Office of Financial Research (OFR FSI) measures global financial market stress. It has both negative and positive values, signifying increasing market stress as the index rises. OFR FSI is used because of its global scope (Monin 2019) and daily frequency, fully matching the wavelet application. The source of data is the Office of Financial Research (2020).

The Morgan Stanley Capital International (MSCI), retrieved from Thomson Reuters Eikon, is used as a proxy for the economic pulse, measuring the global economic activity as a stock market index from companies throughout the world. Energy stocks represent a share of 3 to 4% of the MSCI’s market capitalization at the time of the data gathering.^1^

Additionally, the two interacted variables are constructed to capture the market stress and global economic activity, both under the pandemic context: (i) the interacted pandemic and OFR index and (ii) the interacted pandemic and MSCI index. They are obtained as a product between the pandemic signal and OFR index, pandemic signal and MSCI index, respectively.

All variables are treated in their natural logarithm forms. Having also negative values, the OFR index is rescaled by adding 5.4 without affecting its statistical distribution (i.e. minimum historical level – not covered in our study – is − 5.334, being registered on February 22, 2007). In this way, the adjusted OFR time series has strict positive values allowing it to calculate its logarithm form. We note that all variables are considered at their level as the stationary property is not required in wavelet analyses (Aguiar-Conraria et al. 2008). The descriptive statistics of basic raw variables are presented in Table 1.

### Methodology

The wavelet methodology is employed to study the interaction between oil energy and renewable energy under pre-, first wave and recovery summer pandemic crisis over the period July 01, 2019–June 26, 2020. Unlike the classical time-domain econometrics methods, a wavelet is a time–frequency approach offering results of better quality. To support this, cited by Rua (2010), Clive Granger shows that it is impossible to exhibit economic variables with the same relationship at all frequencies. Moreover, the wavelet tool compactly reveals not only the sign, intensity and direction of co-movement between variables but also how those evolve at different frequencies over time.

The core element in the wavelet analysis is the wavelet transformation. It is a step in which the time series are converted into time-frequencies through a wavelet function (e.g. Haar, Morlet, Mexican hat, Paul or Daubechies). As the Morlet wavelet noted, ${\psi }_{0}\left(\eta \right)$ offers ‘a good balance between time and frequency localization’ (Grinsted et al. 2004, p. 563); this function is finally considered in our approach as follows:

1 $${\psi }_{0}\left(\eta \right)= {\pi }^{-\frac{1}{4}}{e}^{i{\omega }_{0}\eta }{e}^{-\frac{1}{2}{\eta }^{2}},$$

where *η* represents the nondimensional ‘time’ parameter, *ω*_*0*_ is the nondimensional frequency, while *i* stands for $\sqrt{-1}$. The frequency is set to 6, following in this way the admissibility condition suggested by Farge (1992).

Based on the Morlet wavelet, the continuous wavelet transformation (CWT) of a discrete time-series {*x*_*n*_} in both time (*δt*) and scale (*s*), with *n* = *0, 1,…, N − 1* and *m* = *0, 1, …, N − 1*, has this shape:

2 $${w}_{n}^{x}\left(s\right)=\frac{\delta t}{\sqrt{s}} \sum\nolimits_{n\prime=0}^{N-1}{x}_{n\prime}{\psi }^{*}\left(\left(n\prime-m\right)\frac{\delta t}{s}\right).$$

Once transformed, the co-movement between two time series *x* = {*x*_*n*_} and *y* = {*y*_*n*_} can be analysed by using the wavelet coherency tool, including their phase difference (Torrence and Compo 1998; Grinsted et al. 2004; Ng and Chan 2012).

The *wavelet coherency* (*WTC*) is an adjusted cross-wavelet spectrum (*XWT*) developed by Hudgins et al. (1993). The XWT connects two CWT time series ${W}_{n}^{x}$ and ${W}_{n}^{y}$ as follows:

3 $${W}_{n}^{xy}={W}_{n}^{x}{W}_{n}^{y*},$$

the cross-wavelet spectrum being obtained as $\left|{W}_{n}^{xy}\right|$.

Unfortunately, the XWT ‘can show strong peaks even for the realisation of independent processes suggesting the possibility of spurious significance tests’, as Aguiar-Conraria and Soares (2011, p. 649) noted. Therefore, the WTC has been developed to fix the aforementioned issues, considering the smoothing operator *S* for both time and scale, with this form:

4 $${R}_{n}\left(s\right)=\frac{\left|S\left({s}^{-1}{W}_{n}^{xy}(s)\right)\right|}{S{\left({s}^{-1}\left|{W}_{n}^{x}\right|\right)}^\frac{1}{2} S{\left({s}^{-1}\left|{W}_{n}^{y}\right|\right)}^\frac{1}{2}}$$

Additionally, the WTC allows the analysis of the phase angle or phase difference, where the position in the pseudo-cycle of a time series *x* = {*x*_*n*_} is noted *ϕ*_*x*_. For two time series *x* = {*x*_*n*_} and *y* = {*y*_*n*_}, the phase difference becomes ${\phi }_{x,y}\in \left[-\pi , \pi \right]$ as follows:

5 $${\phi }_{x,y}={tan}^{-1}\left(\frac{\mathfrak{I}\left\{{W}_{n}^{xy}\right\}}{\mathfrak{R}\left\{{W}_{n}^{xy}\right\}}\right),$$

where the real and imaginary parts of a complex number are ℜ and ℑ, respectively. Herein, *x* leads *y* when ${\phi }_{x,y}\in \left[0, \frac{\pi }{2}\right],$ while *y* leads *x* for ${\phi }_{x,y}\in$ $\left[-\frac{\pi }{2}, 0\right]$. Conversely, *x* leads *y* for ${\phi }_{x,y}\in \left[-\pi ,-\frac{\pi }{2}\right]$, and *y* leads *x* when ${\phi }_{x,y}\in \left[ \frac{\pi }{2}, \pi \right]$. Time series are considered to be in phase when the phase difference is equal to zero, and in anti-phase for phase difference *π* or –*π.*

**Fig. 1:**
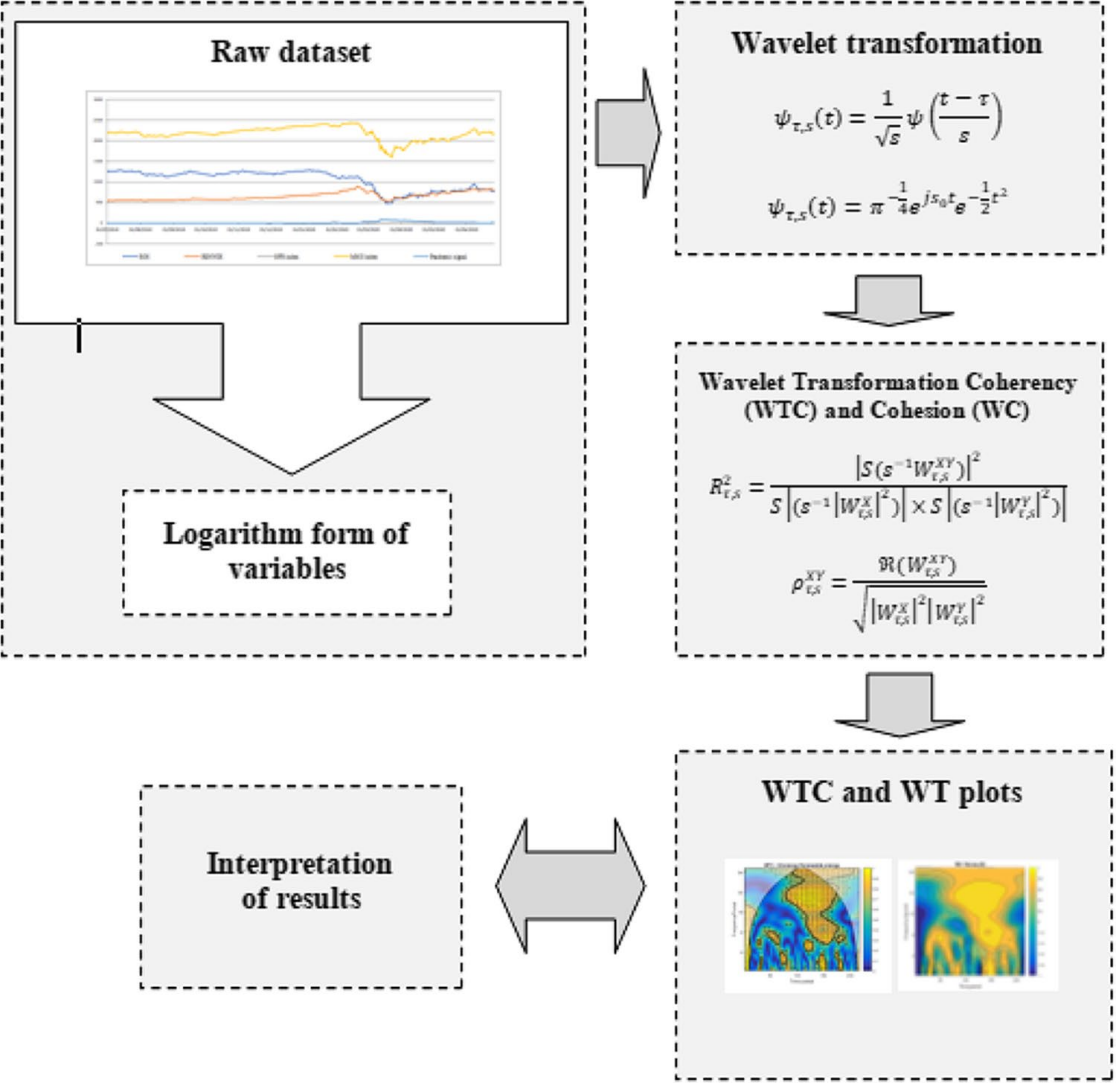
Flowchart of wavelet methodological process. Source: adapted based on Mutascu et al. (2022, p. 9)

For better comprehensiveness, the whole methodological process is graphically represented in Fig. 1, sequentially including dataset treatment (i.e. rescaled for comparability by using logarithm form), wavelet transformation, wavelet estimations and final interpretation of generated results.

## Findings

**Fig. 2:**
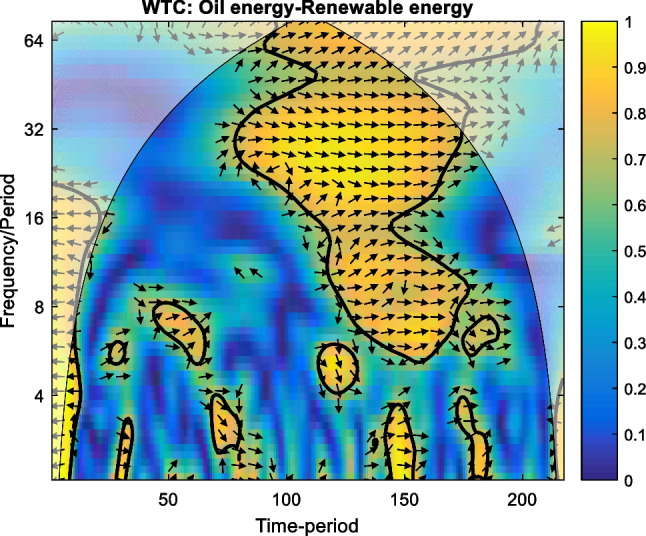
WTC for oil energy–renewable energy. Note: (1) By following the phase-randomised surrogate series, the thick black contour depicts the 5% significance level estimated by the Monte Carlo simulations. The lighted colour shows the areas outside of the cone of influence where the edge effects are biased. (2) The colour code for co-movement power goes from blue (low power) to yellow colour (high power). (3) The variables register cyclical effect in the phase (i.e. arrows are pointed to the right) and anti-cyclical effect in anti-phase (i.e. arrows are oriented to the left). (4) The oil energy is leading when the arrows are pointed to the right and up or to the left and down, respectively. Renewable energy is leading when the arrows are pointed to the right and down or to the left and up, respectively. (6) Legend for horizontal axe: 50 – 20/09/2019; 100 – 27/12/2019; 150 – 12/03/2020; 200 – 29/05/2020

The WTC plot of the interaction between oil and renewable energy is presented in Fig. 2 below. We can see that at high frequency (i.e. short term), for up to 4 days band of scale, the co-movements between oil and renewable energy are rather idiosyncratic, showing isolated episodes of co-movements. By contrast, a period of intense co-movements is registered on medium-to-low frequencies (i.e. medium-to-long terms, for more than 7 days band of scale) between November 22, 2019, and April 04, 2020. Herein, the arrows are pointed to the right, indicating that the variables are in phase, having the same sign. Initially, as the arrows are oriented to the right and up, the oil leads renewable energy, between 7 and 14 days band of scale. Furthermore, between 14 and 48 days band of scale, the arrows are pointed to the right but down, which signifies the renewable energy leading. Finally, for more than 48 days band of scale, the arrows are oriented to the right and up, which means the oil energy is leading again in this band of scale.

Very interestingly, the plot reveals that the co-movement oil energy–renewable energy is exclusively registered only during the first wave of the pandemic crisis, from late November 2019 to April 2020. This suggests, in line with the literature, a clear ‘decoupling’ effect before and after the first wave of disease. The connection between oil energy and renewable energy in low frequencies, which signifies the long-term connection, operates only during the first ‘core’ of pandemic turbulence. Moreover, this clear correlation between oil and renewable, with oil as the leading commodity, shows the still important role of oil energy as a marker for the energy market as a whole.

**Fig. 3:**
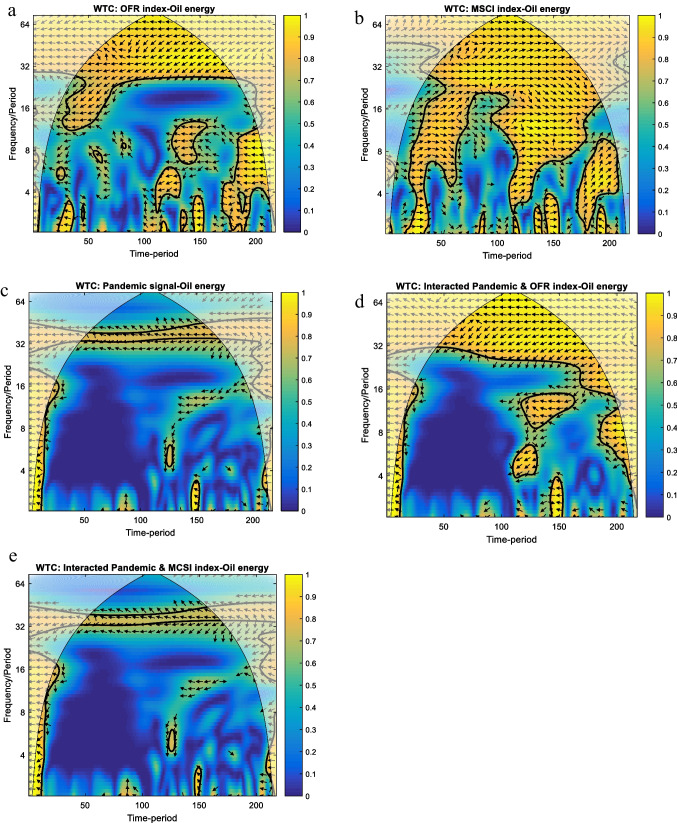
WTC results for oil energy and analysed variables. Note: (1) For interpretations, please refer to Fig. 1. (2) Legend for horizontal axe: 50 – 20/09/2019; 100 – 27/12/2019; 150 – 12/03/2020; 200 – 29/05/2020

The reaction of oil energy under pre-, first wave and recovery summer context is explored in parallel with the OFR index and global MSCI index as well as they are related to pandemic interaction (i.e. interacted pandemic and OFR index – oil energy; interacted pandemic and MSCI index – oil energy). The main outputs are presented in Fig. 3.

The further set of figures looks at the relationship between oil energy and other analysed variables. Figure 3a generally shows that the OFR index negatively leads oil energy for more than 30 days band of scale, the arrows being pointed to the left and down. A significant episode is registered in the short term, up to 14 days band of scale, over April 04–May 25, 2020. In this case, the arrows are oriented to the left and up, suggesting that the oil energy negatively runs the OFR index. These results allow the conclusion, in line with the literature, that oil energy is strongly influenced by what is happening in the financial markets, i.e. a higher ‘financial stress level’ has a negative effect on the oil energy market. The assumption of a strong connection is further strengthened by the second episode, confirming the results presented by Baumeister and Kilian (2016). During this period, the price of oil fell to partly negative values. The financialization of the commodity markets mentioned in the literature reveals that exceptionally oil price hikes would be accompanied by a collapse of financial markets. The connection is invoked by an indissoluble link between oil prices and financial market stress (Ciner 2001; Zhang 2017; Yao and Kuang 2019; Chen et al. 2014; Cunado and de Gracia 2014; Baumeister and Kilian 2016). This is exactly what our results show.

The oil energy positively leads the MSCI index for more than 30 days band of scale, the arrows being pointed to the right and down, as Fig. 3b reveals. Two similar extensions of this co-movement are evidenced in short-to-medium terms, up to 30 days band of scale, between August 05–November 11, 2019, and January 29–May 25, 2020, respectively. Unlike those episodes, the MSCI index positively leads oil energy between April 22, 2020, and May 25, 2020, up to 10 days band of scale, the arrows being pointed to the right and up. The interesting aspect of this result is that the oil energy market is obviously reacting faster than the overall stock market. The exception is again the extreme episode in April–May.

In general, the pandemic signal is not related to the oil energy (see Fig. 3c); the only exception is found for the 32–34 days band of scale, the arrows registering a left to up orientation, implying oil was falling when pandemic signal strengthened (oil is leading, but this can be due to Covid-19 incubation time and its dynamics). A similar finding reveals Fig. 3e devoted to the link between the interacted pandemic and the MSCI index and oil energy. Furthermore, the interacted pandemic and OFR index negatively leads oil energy for frequencies above 30 days band of scale, as Fig. 3d illustrated (i.e. arrows are pointed to the left and down).

**Fig. 4:**
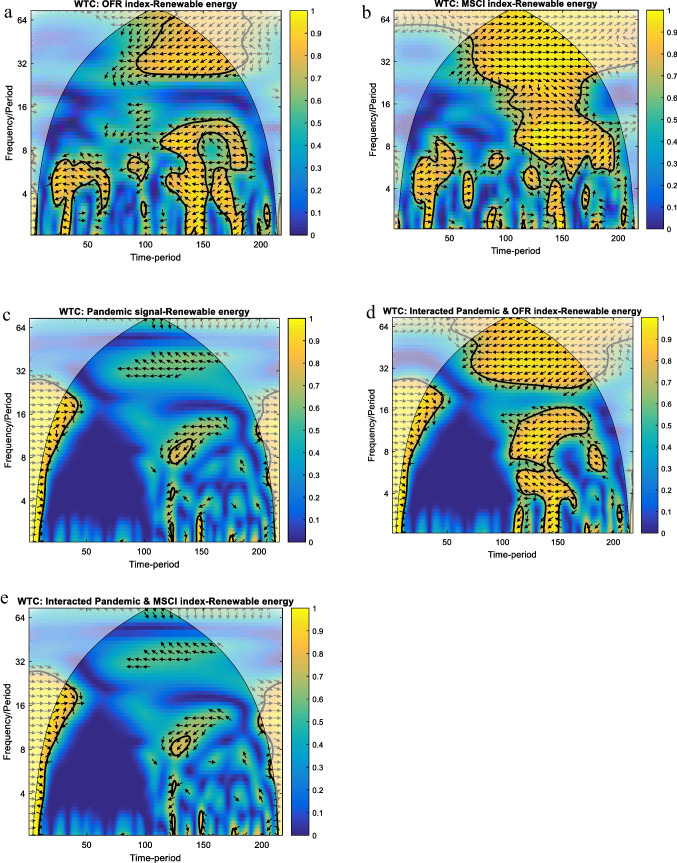
WTC results for renewable energy and analysed variables. Note: (1) For interpretations, please refer to Fig. 1. (2) Legend for horizontal axe: 50 – 20/09/2019; 100 – 27/12/2019; 150 – 12/03/2020; 200 – 29/05/2020

Figure 4 shows the WTC plots of renewable energy’s reaction under pre-, first wave and recovery summer context, in parallel with the OFR index and the global MSCI index as well as their related interactions with pandemic disease (i.e. interacted pandemic and OFR index – renewable energy; interacted pandemic and MSCI index – renewable energy).

Figure 4a indicates that the OFR index negatively leads renewable energy above 30 days band of scale over the period December 27, 2019–May 29, 2020, the arrows being oriented to the left and down. Additionally, the other two episodes are eloquent: over July 29, 2019–October 17, 2020, for up to 7 days band of scale, and January 29, 2019–May 14, 2020, for up to 14 days band of scale, respectively. In the first episode, the renewable energy negatively runs the OFR index (i.e. arrows are pointed to the left and up), while in the second one, the OFR index negatively leads renewable energy (i.e. arrows are pointed to the left and down). At the peak of the crisis, there is a correlation between financial stress and renewables, which vanishes before and after the crisis.

Furthermore, Fig. 4b reveals that renewable energy positively runs the MSCI index (i.e. arrows oriented to the right and down), the direction inverting as the frequency increases (i.e. arrows oriented to the right and up). In this case, the co-movement is significant up to 7 days band of scale, over November 11, 2019–April 29, 2020.

Both Fig. 4c, d, as expected, point out no relevant co-movements between pandemic signal and renewable energy, and interacted pandemic and MSCI index and renewable energy, respectively. Figure 4e shows quite similar results regarding the interacted pandemic and OFR index as for the OFR index in Fig. 4a.

All in all, the findings suggest that the financial signal and economic global activity are significantly interlinked with oil energy in the long term. The pandemic crisis has just a short ‘impulse’ on the oil energy, being more important when it is overlaid with the financial signal. Unlike oil energy, renewable energy seems to be less connected with financial stress, except from the peak of the Covid-19 pandemic. On the other hand, renewables seem to be correlated, especially with economic global activity during the first wave of pandemic disease, shown by the comparably high degree of co-movement in this time, compared with pre-crisis co-movement. Isolated, the pandemic signal has no effect on renewable energy but can be ‘activated’ only under a financial signal.

The results also indicate that the ‘coupling’ effect between oil energy and renewable energy is evidenced only under the first wave of the pandemic crisis, the economic global activity having very important implications than both pandemic and financial signals. Therefore, a high propensity for renewable energy is registered during the first wave of the pandemic disease to the detriment of the oil one.

## Robustness check

We conduct three robustness checks. First, we investigate the ‘renewable–oil energy’ nexus for a much longer period, covering the timespan from January 25, 2007, to June 26, 2020. By investigating this extended period, we can make additional developments visible and thus also check whether our assumptions on decoupling pre-pandemic are tenable. This assumption is related to the contributions of Khan et al. (2017), claiming no significant interaction between oil and renewable energy sector. Such a link is invoked by the interaction between oil energy and renewable energy, especially under specific shocks.

Second, we complement our robustness analysis by applying an alternative wavelet tool to WTC, namely wavelet cohesion. The *wavelet cohesion (WC)* is proposed by Rua (2010), having its root in the contribution of Croux et al. (2001). Comparing to WTC, the WC offers information about the phase of two time series with more accuracy, unfortunately failing to capture also its related lead-lag status.

By using only the real part of wavelet cross-spectra, Rua’s (2010) co-movement measure ${\rho }_{{x}_{n}{y}_{n}}$ is a real number on [− 1, 1], as follows:

6 $${\rho }_{{x}_{n}{y}_{n}}=\frac{\mathfrak{R}({W}_{n}^{x}{W}_{n}^{y})}{\sqrt{{\left|{W}_{n}^{x}\right|}^{2}{\left|{W}_{n}^{y}\right|}^{2}}}.$$

Correspondingly, the WC has the capacity to capture not only the positive co-movements between variables but also those negative ones.

Third, both WTC and WC methods are employed to analyse the ‘renewable–oil energy’ link over the extended period by using monthly frequency. The dataset is obtained as a monthly average of the daily span.

**Fig. 5:**
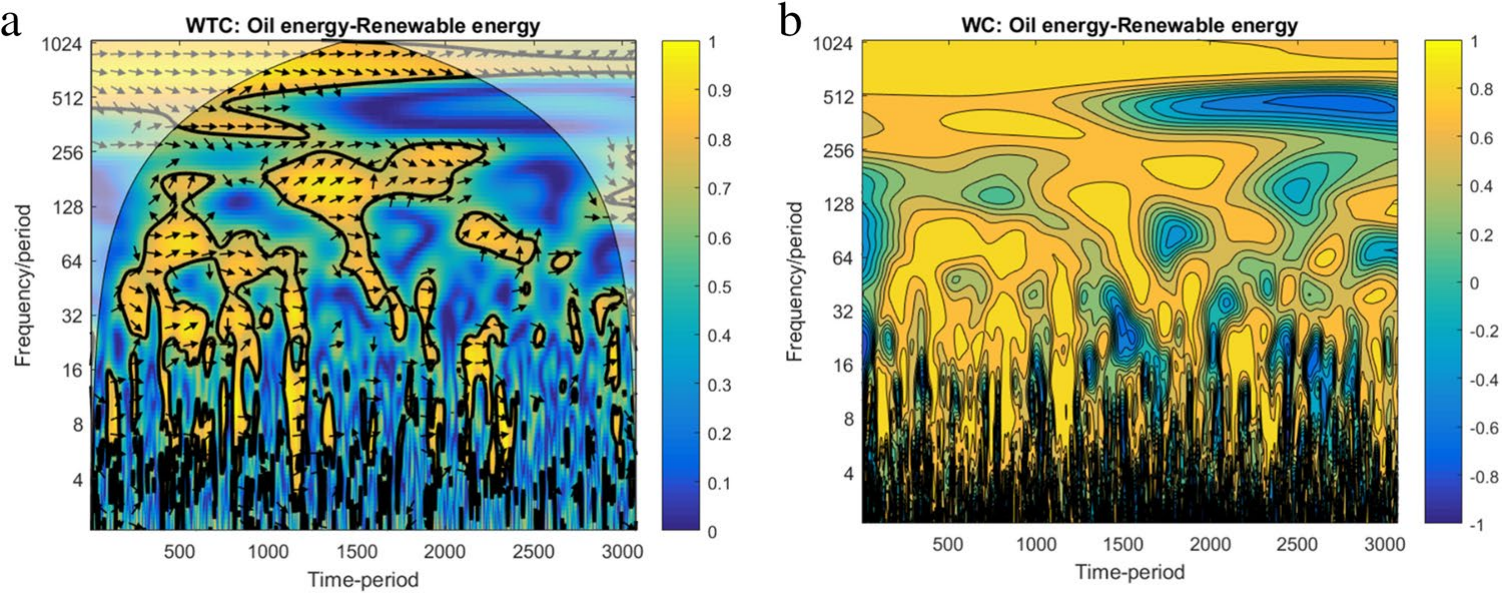
WTC and WC for oil energy–renewable energy for an extended period. Note: (1) Legend for horizontal axe: 500 – 03/02/2009; 1000 – 07/02/2011; 1500 – 12/02/2013; 2000 – 25/02/2015; 2500 – 01/02/2017; 3000 – 06/03/2019. (2) The colour code for the WC on the left side shows both direction and intensity of co-movement, ranging from blue (positive co-movement) to yellow one (negative co-movement)

- (1) The WTC and WT of oil energy–renewable energy nexus on the extended period are plotted in Fig. 5.

Both plots reveal that, at low frequency, for more than 128 days band of scale, significant changes in the co-movement between 2015 and 2017 are observed. On this long-term scale, we can find strong decoupling processes. The start of those processes is interestingly located close to the Paris Climate Agreement, which was signed on December 12, 2015. Therefore, a significant policy-induced decoupling process pre-pandemic seems likely. Otherwise, at high frequency, up to 30 days band of scale, the co-movements between oil energy and renewable energy are rather idiosyncratic.

**Fig. 6:**
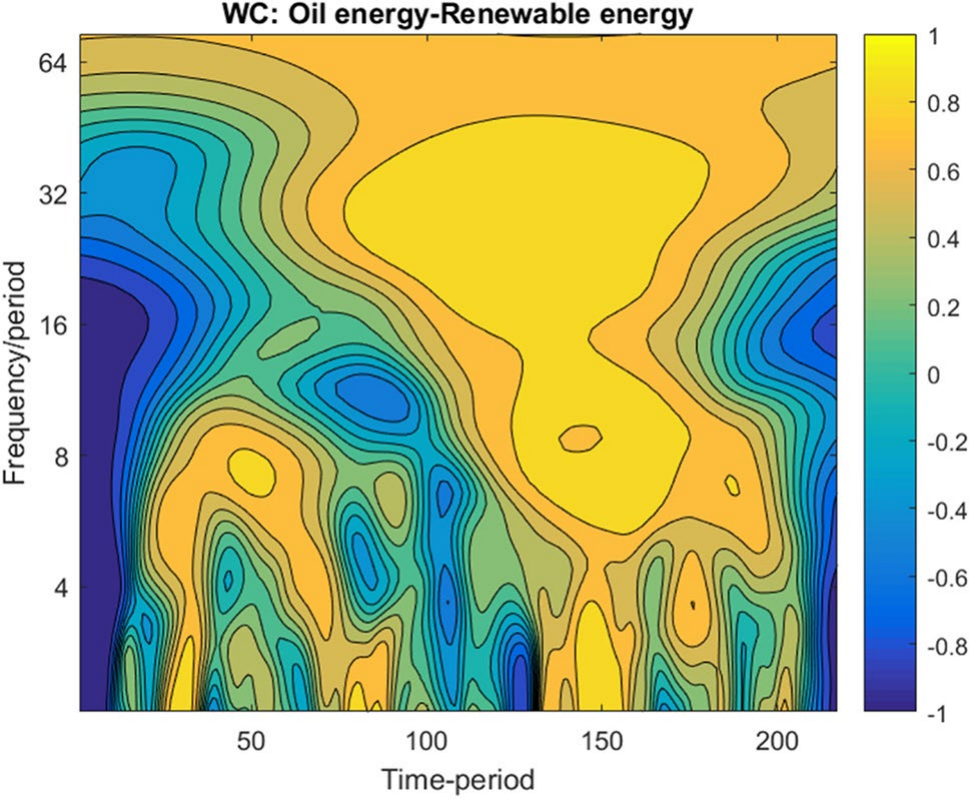
WC for oil energy–renewable energy. Note: (1) For interpretation, please refer to Fig. 4. (2) Legend for horizontal axe: 50 – 20/09/2019; 100 – 27/12/2019; 150 – 12/03/2020; 200 – 29/05/2020

- (2) Fig. 6 plots the WC of oil energy–renewable energy nexus for the initial period scenarios, more precisely over July 01, 2019–June 26, 2020.

The WC plot clearly shows that starting with high to low frequencies, from late November 2019 to April 2020, when the pandemic disease registered its first wave pick, significant interactions are observed between oil energy and renewable energy. The intense yellow colour indicates that, in this pandemic episode, the oil energy and renewable energy co-move with the same sign. Otherwise, their co-movements are rather chaotic at high, medium and low frequencies, the WC showing a mix of changing colours.

Therefore, the WC plot partially reinforces the WTC outputs, validating without any doubt the coupling effect between oil energy and renewable energy during the first wave of the pandemic disease (yellow areas). Regarding the decoupling effect before and during the recovery summer of the pandemic crisis, it is not conclusive as the plot shows an alternation of both positive and negative co-movements between variables.

**Fig. 7:**
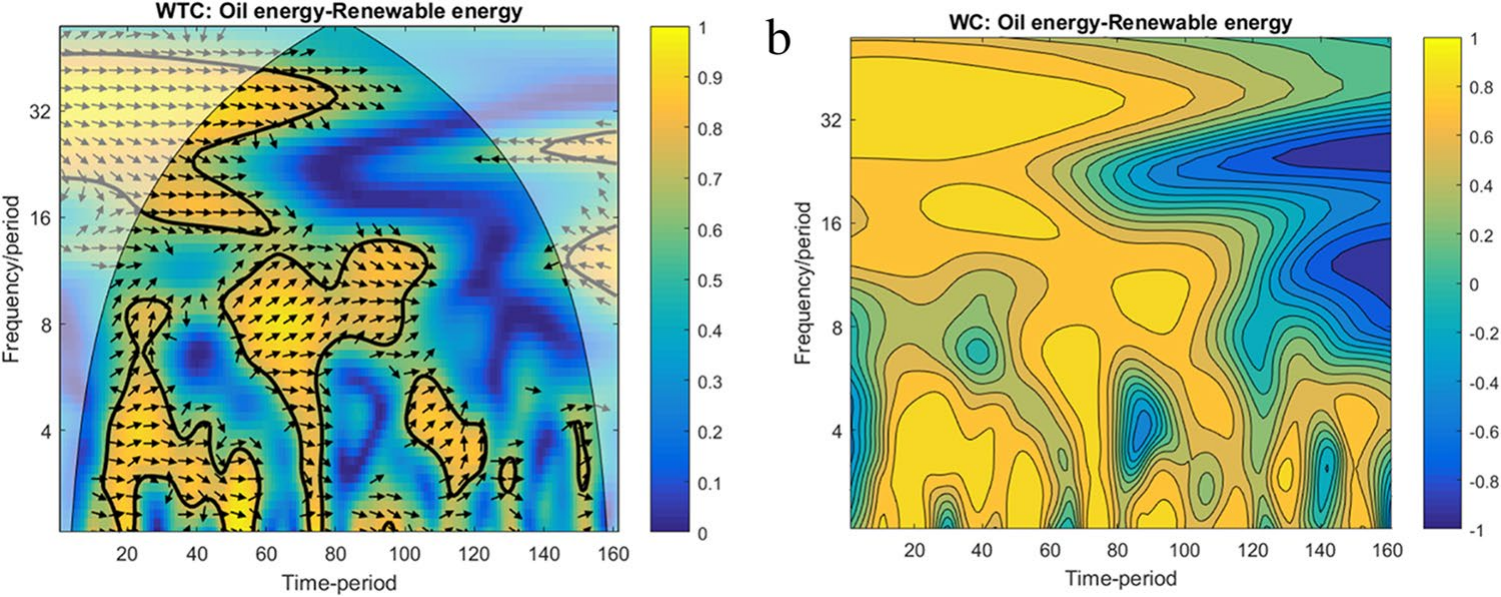
WTC and WC for oil energy–renewable energy for an extended period (monthly frequency). Note: (1) Legend for horizontal axe: 20 – 07/2008; 40 – 05/2010; 60 – 01/2012; 80 – 09/2013; 100 – 05/2015; 120 – 01/2017; 140 – 09/2018; 160 – 05/2020. (2) The colour code for the WC on the left side shows both direction and intensity of co-movement, ranging from blue (positive co-movement) to yellow one (negative co-movement)

- (3) The WTC and WT of oil energy–renewable energy nexus over the extended period with monthly frequency are plotted in Fig. 7.

Both WTC and WC plots reveal almost the same results as WTC and WC in Fig. 5a, b, reinforcing the robustness of the findings. The apparent differences come from frequency, monthly estimations enlarging a lot the plot scale in parallel with the diminution of image resolution (i.e. from daily to monthly frequency).

Corroborating with all the findings, the decoupling effect is evidenced before and after the first wave of the pandemic crisis, validating that the first wave of pandemic disease has the propensity to couple oil energy with renewable energy. The results are in line with Song et al. (2019), Reboredo (2015) or Zhang and Du (2017) as the oil energy seems to be ‘coupled’ with renewable energy under the pandemic shock when the oil price had high volatility. Our results do not confirm Norouzi et al.’s (2020) contributions, who claim that the renewable sector is less affected during the pandemic disease due to its decentralised characteristics.

## Conclusions

The paper investigates the interaction between oil energy and renewable energy markets over July 01, 2019–June 26, 2020, using a wavelet approach. In parallel, pandemic signals, market stress and global economic activity are added as main flavours to this nexus.

The increasing popularity of movements such as *Fridays for Future* demonstrates a high degree of awareness of necessary changes towards sustainable economic development and especially towards the use of renewable energy sources. However, there is less agreement on how to achieve the necessary investments. Comparably low and highly volatile oil prices have made renewable projects both highly risky and unprofitable. Moreover, there has been little latitude for global cooperation in this area so far. As a result, renewable investments represented isolated niche products, as confirmed by our results. We provide evidence for a decoupling effect between oil energy and renewable energy before 2020, as is already suggested by further literature.

The Covid-19 crisis may have changed this situation significantly. The changes in production structures seem to reflect expected global changes in environmental protection, which are further supported by the necessary and generous fiscal programmes.

Firstly, our results confirm a connection between oil and financial market. Moreover, there is an emerging connection between renewables and financial market during the crisis. On the one hand, this seems to be a side effect of the more complex financialization of oil compared to the less institutionalised renewable energies. On the other hand, it may reflect the restructuring towards alternative energy sources. In any case, it is a strong mutual relationship. As a rule, the price of oil seems to react to financial upheavals, but it can also itself become a source of stress in the financial markets, as the episode in April/May 2020 shows. Moreover, the Covid-19 crisis shows that with increasing institutionalisation, the first connections and reaction mechanisms are also emerging between renewable energies and financial stress. Somewhat ironically, the period of high fossil energy prices, together with sanctions and supply barriers due to the Ukraine war, can further strengthen this process.

Secondly, we demonstrate that the first wave of the pandemic disease had the propensity to couple oil energy with renewable energy. However, the co-movement of oil and renewables can only be seen for a short period, the heavy impact period during the first wave of the Covid-19 pandemic. It seems that low energy prices are still an important counterfactor for investments in renewables. On the other hand, the decoupling effect already found in the literature now seems to be sustainable enough for renewable energies to develop independently of the oil market, which is the first major step towards a sustainable economy from a macro-financial perspective.

Thirdly, the renewable energy and oil markets have a different relationship with the general global economic activity. This can be based on two mechanisms. On the one hand, renewable energies could be irrelevant due to their market share, so their performance is not reflected in the overall economic development. On the other hand, the development of renewable energies may also be determined by social and political factors, which leads to independence from economic development.

Therefore, our findings evidence that pandemic disease is veritable glue for oil–renewable energy nexus, market stress and global economic activity also having significant complex implications. The main policy implications of results are related to adjustments related to the more general renewable energy area, subsidiary also covering the behaviour of the agents on the capital markets.

1. Regarding the energy area, two major policy directions can be derived from our results: one is purely political, while another is an institutional one. First, from a political perspective, the decoupling of renewable energies from the oil market should be accelerated by identifying clear use cases for renewable energies. As already mentioned in the literature, therefore, it might be useful to point out the different fields of application of oil and renewable energies. Of course, political support, for example through the Paris Climate Agreement, for a sustainable energy economy is also part of this point.

Secondly, the institutionalisation and financialization of renewable energies should be promoted. The negative aspects, such as a stronger link to financial and market stress, should be offset by positive effects such as investor attention, security and stronger diversification and hedging opportunities.

(2) We can also identify two major policy directions for agents in the capital markets. First, for investors, a mix of renewable energy stocks and fossil fuel energy stocks could help to diversify portfolio risk. Thereby, two models of action occur. On the one hand, the decoupling of renewables and fossil energy stocks diversifies the risk, and on the other hand, the inclusion of renewable energy stocks reduces the risk of negative publicity. Last but not least, active trading in the financial markets can also improve the basic liquidity of renewable energy firms and thus the ability to act in the field.

Further research should be developed by taking into account, on the one side, the next pandemic disease waves and the consequences of the war in Ukraine, and on the other hand, an extended pallet of economic determinants as soon as their related datasets will be officially available.

## Data Availability

All data are taken from publicly available sources. The datasets used and/or analysed during the current study are available from the corresponding author upon reasonable request.

## Appendix

See Table 1

**Table 1:** Descriptive statistics of raw variables

Variables	XOI	RENIXX	OFR Index	MSCI Index	Pandemic signal
Mean	1044.571	665.6674	− 0.625880	2180.218	14.71889
Median	1169.880	642.1200	− 2.132000	2201.140	0.000000
Maximum	1293.410	893.0400	10.26600	2434.950	98.00000
Minimum	469.6800	525.8100	− 4.242000	1602.110	0.000000
Std. dev	239.1842	95.08849	3.627894	166.9983	23.14546
Skewness	− 0.739249	0.487861	1.265436	− 0.935199	1.837117
Kurtosis	2.012016	1.955818	3.540396	3.883153	5.625754
Jarque–Bera	28.59035	18.46623	60.55515	38.68342	184.4010
Probability	0.000001	0.000098	0.000000	0.000000	0.000000
Sum	226,671.9	144,449.8	− 135.8160	473,107.2	3194.000
Sum sq. dev	12,357,158	1,953,033	2842.909	6,023,902	115,713.9
Observations	217	217	217	217	217

## Footnotes

1 Its composition can be found in the MSCI World Index fact sheet.
